# Supplementary material for: Meaningful everyday life situations from the perspective of children born preterm: A photo-elicitation interview study with six-year-old children
Source: PLoS One. 2023 Aug 14;18(8):e0284217. doi: 10.1371/journal.pone.0284217 (PMC10424858; doi:10.1371/journal.pone.0284217)
Supplement: S1 Table — (PDF) [file pone.0284217.s001.pdf]

S1, Table. Generic category, Significant circumstances to do things

Condensed meaning unit	Code	Subcategory
B90: To have a favorite list including the family and relatives	Having parents, family, relatives mother and father-parents, girlfriend and animals who can do different things such as comfort, set boundaries, take care, give good advice climb, dance ballet or do things for me (sewing dress-up clothes, carpenters bed), are important and that I/man love are valuable and with which I feel safe.	To have significant others
B160: mother, father and my brother are important		
B20: To have a girl friend who climbs (the climbing walls) to the top and who dances ballet		
B130: To be with my mother and do a lot of things together and she comforts me when I'm sad, because I love her, she is like med and she loves me		
B130: To be with my father whom I love and who is wise, smart, practical and gives good advice, good and valuable. I like everything about my father.. feel safe		
B40: To know a dog that doesn't live nearby		
B130: To sit by myself on the couch and watch TV, cartoons about fairies with magical powers, on Netflix and the children's channel, and at the same time eating sandwiches made by dad, that I like		
B90: The climbing frame is often used especially during summer and the new part that my father has built. I got the climbing frame as a birthday present. I'm good at climbing, you'll have to do your best. Mother is the ref		
**Condensed meaning unit**	**Code**	**Subcategory**
B10: [To known how to] cutting, taping and gluing together what you have drawn	To be able to [master] one's temperament and/or to know and be good at doing different things such as: cutting, taping	To have significant skills
B140: To be able to do high speed in the swing "Green face"		

S1, Table. Generic category, Significant circumstances to do things

B10: [it's important to know how to draw]. [You'll have to practice] to learn how to draw as good as I do. I practice drawing	and gluing together what one has drawn, doing high speed on the spinning swing, drawing, building a boat, daring to run and jump on the trampolines, pancake batter and cracking eggs, diving and swimming underwater, playing outside, swinging and climbing trees, building ships and airplanes, with legos, looking at the moon, stars, sun, planets and milky way with my binoculars, they can be hard to find but I know a lot about them, riding, getting on the horse, skating forwards and backwards that are promoted by specific skills (imagination, freehand, endurance) to achieve a goal such as getting better at something.	
B140: To be at the Jump yard and to know that I dare to run and jump on the trampolines [is the most fun of all]		
B120: To be able to master your temperament and to build a boat		
B140: To be able to make pancake batter and to crack eggs		
B40: I know how to dive and swim a little but not above the surface because there you have to keep up.		
B120: To play outside, to swing and climb the trees is fun and I'm good at it		
B40: To build ships, airplanes in LEGO I'm good at it and I build without instructions		
B20: To look at the moon, stars, sun, planets and Milky way with my binoculars, they can be hard to find but I know a lot about them.		
B40: To build boats, helicopters and airplanes in LEGO and to build from my own imagination		
B90: To read by myself books like Nelly Rapp and Lasse-Maja. It's good to know how to read. To practice one's voice [when you read] and it's fun with the book shelfs		
B20: To skate forward and backwards I am good at it. Everything is easy except to do bubbles with the skates		

S1, Table. Generic category, Significant circumstances to do things

B160: To be good at riding and enjoying it a lot, I have been riding for a long time. I can get on the smallest horse.		
B10: To play Super-Mario,Wee, with my little brother [is something that] I like and know because I'm good at it		
B10: I like to play Monopoly with everyone and I am good at it		
B130: I'm good at getting out of situations [self-defense] because I trained and practiced until I got better		
B20: To be good at climbing as I have been doing it for a year and can climb near the top. And is important to me. You need small hands to grasp the grips and need to be agile to climb. Outside, I get all the way to the top of a smaller mountain		
B150: To play cards with the family and to play cards in the spare time with friends is fun because you learn a lot and I often win. You have to be smart to be good at card games		
B90: [I have] you need to have speed and strength to be able to stay [on the climbing frame] so as not to fall down and tear yourself		
**Condensed meaning unit**	**Code**	**Subcategory**
B60: To wear nice and fun clothes	To have different things like nice and funny clothes, theatre costumes, raspberry bushes, a safety box for thing you are fond of, a bed of your own	To have significant things
B20: To have raspberry bushes		
B150: To have a safety box with a code where you safely can store things, for example Pokémon cards		

S1, Table. Generic category, Significant circumstances to do things

B90: To have a costume, in which I play, with buttons, lace and flower made by grandmother		
B90: To like my bed made by my grandfather and my father. It's important to sleep otherwise you will not get energy, I get energy at night and like to play. When I can't sleep at night, I get tired during the day instead, that's a small concern, then I rest during the day instead.		
