# Supplementary material for: Meaningful everyday life situations from the perspective of children born preterm: A photo-elicitation interview study with six-year-old children
Source: PLoS One. 2023 Aug 14;18(8):e0284217. doi: 10.1371/journal.pone.0284217 (PMC10424858; doi:10.1371/journal.pone.0284217)
Supplement: S2 Table — (PDF) [file pone.0284217.s002.pdf]

S2, Table. Generic category, Doing things

Condensed meaning unit	Code	Subcategory
B150: To build with machines and space ships in LEGO	Doing fun things like winning things, building with Legos, dressing up, going to the school Halloween party, listening to fairy tales, reading, crafting, snow racing, going on vacation, dancing, giving me five and playing games, buying, saving for and collecting Pokemon cards, reading about Pokemon's characteristics are things that I/I do often and that I/I am good at.<br><br>Doing important things like hugging and helping.	Doing things that are fun and important
B90: To dress and choose a dress, to play in it or to go to a Halloween party at school		
B90: To listen to fairytales on the I-pad is really nice		
B90: To make old men out of toilet paper rolls, balls of wool, pearls, green figures out of clothes are fun		
B40: To ride a snowracer in the hill behind the house		
B40: To enjoy activities with a bit of speed		
B20: To build with LEGO some ninjas, goasts, temples …I love that		
B20: To jump from a rock down in the water		
B120: To put pearls on a peg-board is really fun		
B120: To be on holiday and dancing and giving-me-fives afterwards is hilarious		
B120: To read books is very fun		
B130: [To enjoy] playing games and it is not hard		
B90: Att klä ut sig på Halloween och sminka sig, gillar jag.<br>Dressing up on Halloween and putting on makeup, I like that		
B10: Sitting at my desk cutting and painting Zombies and Lightning McQueen with my pencils is something I like to do, do often and am good at. I always want to draw before we go [somewhere]. Then I hurry [to draw]. To draw several different things that you cut out and glue together becomes [more and bigger] and nicer. I mostly draw myself.		

S2, Table. Generic category, Doing things

B90: To read by yourself books like Nelly Rapp and Lasse-Maja. It is good to know how to read. To practice the voice [when you read] and it is fun with the bookshelves		
B140: To do a salad		
B140: To bake a pizza		
B140: To bake a pizza with ham and cheese, that I like		
B140: To make a dough		
B140: To make the pizza round		
B40: To win a teddy bear on the Christmas fair, which you can have at home		
B90: To hug and to help is most important to me. I have quite a lot of things that are important to me.		
B150: To buy, save and collect Pokémon cards and to look at and read about the characteristics of the Pokémons		
B10: To compete against my little brother in Super Mario-Wee. To have my favourite guy: Super Mario		
B120: To play mother-father-child and to play that you work in a café at school, I like that very much although I’. beginning to get tired on the café and have started to draw		
B130: I like to sit on the couch by ourself and wath TV, cartoons about fairy with magical power, on Netflix and Barnkanalen [Channel for children], and at the same time eat sandwiches		
B90: To make an obstacle run over the water like the one I have seen on You-tube, of your own climbing frame at home and climb from one end to the other by jumping, swinging and stepping on the swings to reach to goal		

S2, Table. Generic category, Doing things

Condensed meaning units	Codes	Subcategory
B90: To dress up together with a friend	To be with others and do fun things like dressing up (F), play (F, S), play games (F, S, P) swim practice (S, C), hockey practice (F, C), taekwondo (Co) visit grandma in the hospital, fool around (P), hugg (P, S, GP), sew (GP), craft (P, GP, S), celebrate Christmas, carv (GP), walk (GP), sing (GP), look for Pokemon (GP) snuggle (P), bake (P, S, GP), cook (P,GP) sing (GP), read (P), cycle in puddles (S), make excursions and trips, visit museum (GP)<br><br>F= friend<br>S= sibbling<br>C= other children<br>P= parent<br>GP= grandparent<br>Co= coach	To do things with significant others
B160: To be with your friend and to play in the classroom		
B150: To enjoy and to want to often play with my father and little brother		
B150: To play cards with the family and play cards in the spare time with friends is fun because you learn a lot and I often win. You have to be smart to be good at card games		
B40: I go to the swimming pool with my sibblings. I have to be in the smaller pool where I can reach to floor. I go there together with my little brother and practice together with other children		
B90: To visit my grandmother at the hospital and to hug is nice		
B90: I like to be with my grandmother and sew and do needlework		
B150: To be with the family who is kind and does funny things. To play a lot with my little brother.		
B150: [It is important] to be with your best friend who is kind and play games play computer games.		
B160: [I enjoy] being with my father's cousin and take photos and talk because she is kind and I like her		
B160: To play different things with my little brother is fun, I can always play with him, of you don't have anyone else		
B160: To be with my kind father and play and fool around [is important to me], sometimes we disagree		
B160: It is [important to me] to be with my mother [since we like to be together] and play and cozy up together		

S2, Table. Generic category, Doing things

B20. Carving spears with a saw is fun because then you can kill mean animals. Grandpa hunts and kills moose.		
B90: To practice Teakwondo twice a week with other children and with a coach who has a blackbelt with yellow ribbons		
B90: To be with your mother and little brother and like to bake buns or gingerbread once a year or six times a year, because it's good and they have fun shapes. Baking different things is fun. Baking is [more fun] than eating. Being able to help properly and pour in everything. When baking, you need to be careful. It is important to be able to bake in order to make coffee		
B90:. I enjoy and love to be with my father and play the guitar because you have to be smart and find out how to do it		
B90: To print pictures of robots, ice creames and food together with my mother and little brother and to color all afternoon		
B90: It is fun when mother and father read Doktor Proktors pruttpulver [book title]		
B40: To enjoy riding the bike trough puddles of water together with my younger brother so that it splashes on the one behind. To suck at lifting your feet by puddles		
B60: I really enjoy celebrating Christmas with Micke, dressing the tree, getting Christmas presents and watching Donald Duck		
B60: To join and play with my little brother. I love him and [let him play with my things] and he's nice to me and then he wants to hug		
B60: To be with my father who is really good because he fools around with me and he cooks really good food		
B60: To make cinnamon dough together with my father		
B140: To bake, do a dough and a smoothie with my grandmother		

S2, Table. Generic category, Doing things

B140: To bake pizza with my mother		
B140: To enjoy fika and to talk and arguing with my big brother in the kitchen		
B140: To sometimes play with my big brother		
B140: To play a lot with my older sister		
B10: To play card ”Finns i sjön” with my family		
B10: I like to play with Duplo, build houses and figures together with my little brother		
B10: Att spela Super-Mario, Wee, med tillsammans med min lillebror [är någonting] jag tycker om		
B10: Att spela Monopol med alla det tycker jag om		
B60: I like to be with grandma because she gives me toys and candy on weekdays		
B60: To have secret stashes for candy that I can find, along with my grandma		
B60: I like to be with my mother because she is kind and she lets me play on her telephone		
B60: To be with my father, my father is the best, because we me and my brother sometimes are allowed to play on his computer		
B20: To be outside and walking and singing with grandpa wearing a kepa, nearby and holding hands. I usually spend a lot [of time] with grandpa, I like being with my grandpa. We walk every day just for speed, that's why I have strong legs. Now grandpa's leg hurts so I can't walk, then I get to pat his leg and then we get to take a shorter walk. I look for Pokemon and Grandpa watches as I catch Pikachu		
B20: To be with my dog	To be with a pet and do specific things	

S2, Table. Generic category, Doing things

B20: To be with my dog and cuddling and she obeys; lie, walk, sit, walk on hind legs, I like it. The dog accompanies me on my walk with grandpa		
B20: To be with my cat who is kind, beautiful, snuggles and purrs whole time		
B160: Playing with my mother's friend's dog at our house when we are dog sitters as he is kind and happy and you don't have to be afraid, we play with toys and throw things that the dog picks up. The dog is nice and has smooth ears		
**Condensed meaning units**	**Codes**	**Subcategory**
B60: Being in the play corner and cooking pretend food and drawing at school with my friend is the best as I like to cook and want to be a chef	Being in a specific place (the play corner at school, the music room, the kitchen at home, the space room, the studio at school) with a specific person (friend, music teacher, mother, the leader, everyone else, friends) and doing different things that I enjoy (cooking pretend -food, dancing, playing, making music, singing, exercising, drawing) that go with the place and the person	To do things in significant places or contexts
B60: I like being in the music room and having music and playing the piano with Gunilla, her assistant Kasongo and all the other children and dancing, playing, making music and singing		
B140: To be with mother in the kitchen doing things		
B120: To be in the space room and play spies in space going to earth, in school with friends is a lot of fun.		
B120: Being with many [friends] in the studio at school and drawing, that's the most fun		
B120: To be at the museum and build a Viking boat with dad and big sister, it was fun and easy and now it's in the game room at school and I don't want it destroyed		
B20: To climb high up in the tree at school with my girlfriend and to sit on a branch and eat apPles in summer – I like that		
B160: Being with my friend in my classroom and playing with sand that is warm and we do figures and you have to wash your hands after	To be with person (girlfriend, friend) in a specific place (cla and do specific things (play with sand, family, cards)	
B160: Playing ”family” with my friends in her classroom at breaks at school		
B130: To enjoy snuggling in bed		To do things in

S2, Table. Generic category, Doing things

B90: I think it is nice to lay down and to wear my pyjamas and the teddy bears and to sleep particularly with my star with remote control and that can shine in many colours	Being home and snuggle in bed, in kitchen at home [bake, fika, talk], dressing up at home, sitting at the desk at home	significant places or contexts
B140: To be in the kitchen with my family I think baking is fun		
B130: To be in my room, where you can do many different things, with my furniture and all the things I think is cozy and feels safe. [It is important] for me to watch movies, lie down and rest and talk with others or alone, in peace from others. Everyone can be in my room	Being at a specific place [my room] and do things [snuggle, watch TV, rest, talk]by myself or together with others	
B60: To play X-box at grandma's is fun, because there you can play many games, even war games, because I'm good at it, I've learned how the game works and I've had it for a long time	Being at grandma's playing X-box	
B20: To be at school in our classroom in our courtyard	To be at school: the classroom (do homework, play), the LEGO corner, the gym (play, play basketball, killerball) the park, the drama room (play), the school yard (jump, dance, sing), the puppet corner (play), the school library (borrow books)	
B40: To be at the LEGO place at school		
B40: Doing gymnastics at school dressed in gymnastics clothes, shoes, shorts and a t-shirt with a animal on. Having a towel to dry oneself and to shower if you get sweaty		
B140: To swing in the parc at school		
B120: To beg at the drama and play mother-father-child and cat or dog is fun but not so important		
B120: To be in the gymnasium and [doing] different things and playing the tail game, playing basketball I think is a lot of fun		
B40: To play Killerball on the gymnasium is fun. I have many friends that I play with. When school is over, I will start first grade and then I can start somewhere else for gym outside of school.		
B20: Jumping, dancing and singing standing on a stone at school		
B160: To be in the classroom playing with dolls and play family		
B160: I like to be at school and do homework and play		

S2, Table. Generic category, Doing things

B150: To be at the school library lending books that mum and dad can read to us		
B130: I like to be at school and do a lot of things like present your toy in front of the class and do maths, Swedish, sing and sorting [litter]		
B130: To be at school and at the after-school recreation centre having a teacher who is fooling around, having breaks and being with friends is nice		
B60: I like to be at school and I and my friends are allowed to write on the black board and write words		
B140: To be outside and swing, ride the bike and play is fun	To be outside [swinging, bicycling, playing]	
B140: To be at a gym and learn how to do somersaults	To be at the public baths (swimming practice), gym (vaulting, taekwondo,), the stables (riding, swimming, rummage), school with specific persons and doing specific things.	
B90: To be at the gym practicing Teakwondo twice a week in a group with children and a coach		
B40: To sit on the bench [at the public baths] before jumping in is cold, it's cold in the water too but later it gets warmer		
B40: To be at the public baths swimming is a nice place to be		
B160: To be at my riding school with horses and learn about horses from our riding instructor. I like horses, like to brush not saddle		
B160: To be at the stable with horses, to go to riding camps and to bathe with them, I want to be with horses all day		
B20: To climb at mom's school with mom and play with other kids once a week and is important to me. On a wall you can climb up and down. The wall is high and you can fall down, but then you fly. I have climbed outside and inside. Outside, I get all the way to the top of a smaller mountain.		

S2, Table. Generic category, Doing things

B130: To be in the same place as mom practices Brazilian jiunjutsu and do different exercises with her friends from school and learn a lot of techniques/exercises I think is fun.		
B130: I think it's fun to drive a lot with dad and drive over bumps that make you laugh and make your stomach tingle, sit and look out the window, dance, listen to music and play Harry Potter.	To do fun things (bumping, dancing, listen to music, playing) with a significant person (dad) in a special place (in the car)	
B40: To be at the zoo and looking at the monkeys, the show with the delphines. The delphines did trics and somersaults. I like animals	To be at the zoo and look at the animals	
