# Supplementary material for: Meaningful everyday life situations from the perspective of children born preterm: A photo-elicitation interview study with six-year-old children
Source: PLoS One. 2023 Aug 14;18(8):e0284217. doi: 10.1371/journal.pone.0284217 (PMC10424858; doi:10.1371/journal.pone.0284217)
Supplement: S3 Table — (PDF) [file pone.0284217.s003.pdf]

S3 Table. Generic category. Desire for significant development

Condensed meaning unit	Code	Subcategory
B40: To want a pet because you like animals and can take care of them	To want to have and to be with a pets, goats, horses and other animals	To have desire for significant others
B160: To want a horse of your own in the stable and goats that are smooth		
B160: I would like a dog. If I had a dog I would play with it a lot let it sleep beside my bed		
**Condensed meaning unit**	**Code**	**Subcategory**
B140: Wanting to know how to make pancakes	Wanting to know or want to get better at making pancakes, swimming, cutting, do gymnastics, building with Kapla blocks, bicycling without support wheels	To have desire for significant skills
B40: To practice swimming		
B40: To want to get better at cutting black lines on paper, to have your own scissors. (Being disappointed when you couldn't cut out your figures that you drew). You can get better at cutting with practice		
B40: To want to get better in building rotating stairs and with Kapla-blocks. Being at school and building with Kapla blocks together with others or by yourself		
B40: To learn how to ride a bike on the lawn without supporting wheels		
B40: To learn to swim, it is difficult to stay above the surface of the water		
B90: Graduating [in Taekwondo] and changing the belt to a different colour. To want to be as good as your coach but behave and not mess around and fight and to do as the coach says during training		
B40: To be better in doing gymnastics.		

S3 Table. Generic category. Desire for significant development

Condensed meaning unit	Code	Subcategory
B90: To dream of attending the climbing club	Wishing to be able to go to the climbing club, play soccer, be oat the gymnasium more often, work with animals, be with horses all day.	To have desire for significant activities
B140: To be able to play soccer		
B120: To like to have more gymnastics. I would like to be there (gymnasium) more often		
B40: To want to work with cattle when you grow old. When me and my little brother are big we will work at a farm with different kinds of animals		
B160: To be with horses all day		
