# Supplementary material for: Meaningful everyday life situations from the perspective of children born preterm: A photo-elicitation interview study with six-year-old children
Source: PLoS One. 2023 Aug 14;18(8):e0284217. doi: 10.1371/journal.pone.0284217 (PMC10424858; doi:10.1371/journal.pone.0284217)
Supplement: Attachment — Submitted filename: Respons to reviewers.docx [file pone.0284217.s004.docx]

PLOS ONE Decision: Revision required [PONE-D-22-19343] - [EMID:c07c3cf037412591]

PONE-D-22-19343

Meaningful everyday life situations from the perspective of children born preterm: A photo-elicitation interview study with six-year-old children

Response to reviewers

Thank you for your interest in our manuscript. We do appreciate the comments from the editor and reviewers. We have now revised the manuscript. The clarifications and revisions according to the comments have been made (see table below).

Yours sincerely,

Anna Karin Andersson

| **Journal requirements** | **Revised version according to comments** |
| --- | --- |
| Please ensure that your manuscript meets PLOS ONE's style requirements, including those for file naming | The manuscript meets PLOOS ONE's style requirements, including those for file naming. |
| In your Data Availability statement, you have not specified where the minimal data set underlying the results described in your manuscript can be found. PLOS defines a study's minimal data set as the underlying data used to reach the conclusions drawn in the manuscript and any additional data required to replicate the reported study findings in their entirety. All PLOS journals require that the minimal data set be made fully available | Due to the ethical approval, it is not possible to make the interviews fully available. However, we have added a table presenting an overview of the data analysis process from Meaning units to Core category. A minimal data set showing all meaning units, codes, subcategories and generic categories (S1, S2, S3) is uploaded as Supporting Information files. |
| Please review your reference list to ensure that it is complete and correct. If you have cited papers that have been retracted, please include the rationale for doing so in the manuscript text or remove these references and replace them with relevant current references. Any changes to the reference list should be mentioned in the rebuttal letter that accompanies your revised manuscript. | The reference list is reviewed and is complete and correct. |
| **Reviewer’s comments** | **Our answer** |
| 1. Is the manuscript technically sound, and do the data support the conclusions? Reviewer #1: Yes | Thank you |
| 2. Has the statistical analysis been performed appropriately and rigorously? Reviewer #1: N/A | - |
| 3. Have the authors made all data underlying the findings in their manuscript fully available?Reviewer #1: No | As the data consist of interviews with children we, for ethical reasons; preserve children’s confidentiality, make data fully available. |
| 4. Is the manuscript presented in an intelligible fashion and written in standard English? Reviewer #1: Yes | Thank you |
| **Reviewer #1.** |  |
| The authors describe well why the child’s perspective is important, but do not specifically explain why exploring such perspectives in children born prematurely but not diagnosed with disabilities is important. | Thank you for your comment. In the Background section we have added arguments for why the perspective of children born preterm not diagnosed with disabilities is of interest. Being born preterm lead to more care and attention and the perception of the child as vulnerable may influence everyday life situations. |
| The section on ethical parameters should be located earlier in the methods section rather than its current location just before the results section. Putting the ethical section earlier informs the reader of Institutional Review Board approval prior to the recruitment of participants where ethical approval is particularly important. | The section Ethical Considerations has been put earlier in the method section. We have clarified that written informed consent was obtained from the parents and verbal consent was obtained from the children. The information letter and the consent letter were included in the ethical approval. |
| The qualitative design is appropriate for this research. Photo elicitation is an important tool for qualitative research with children given the challenges describing their experiences. This is particularly true for children at age six where the childhood perspective and understanding is narrower than seen at an older age. Using photographs as a prompt provides appropriate support for efforts at eliciting descriptions. | Thank you for this comment. We do very much agree with you. Using photo elicitation made it easier for the children to reflect on their everyday situations. |
| The data analysis process is well described. However, what is not included is reference to trustworthiness of the data and triangulation of data collection/data analysis processes. These elements are critical in the discussion of qualitative research where study rigor is a concern. | Trustworthiness is now discussed in the methodological limitations of the study. |
| The results section is well documented – the terms listed in the core category, generic categories and subcategories are logical and seem to be supported by the data as illustrated by quotes embedded in the text. The discussion section expands on the categories identified in the results section and does so well. However, references to support findings are in large part those addressing activities and participation in children with disabilities. There are other existing studies addressing activities and participation in neurotypical children which should be woven into the discussion section. These concerns relate to the need to explain why children born prematurely were chosen for this study. | Thank you for the suggestion to include studies on typically developed children. We have added studies and discuss our results in relation to studies on neurotypical children and children with disabilities. References have been added; Blencowe et al, 2012; Ballantyne et al, 2016; Elo et al, 2014; Rosenberg et al, 2019; Shields et al, 2018. |
| The theoretical perspective, Family of Person-related Constructs (fPRC), is mentioned in the discussion section. Using this model or others to guide the study would have generated a more meaningful background for the paper. | In the Background section we refer to the Bioecological system theory as a base for our understanding of children’s development. We relate to the fPRC also on the background section. |
