# Supplementary material for: Meaningful everyday life situations from the perspective of children born preterm: A photo-elicitation interview study with six-year-old children
Source: PLoS One. 2023 Aug 14;18(8):e0284217. doi: 10.1371/journal.pone.0284217 (PMC10424858; doi:10.1371/journal.pone.0284217)
Supplement: Attachment — Submitted filename: Response to reviewers.docx [file pone.0284217.s005.docx]

PLOS ONE Decision: Revision required [PONE-D-22-19343] - [EMID:c07c3cf037412591]

PONE-D-22-19343

Meaningful everyday life situations from the perspective of children born preterm: A photo-elicitation interview study with six-year-old children

### Response to reviewers

Thank you for your interest in our manuscript. We do appreciate the comments from the editor and reviewers. We have now revised the manuscript. The clarifications and revisions according to the comments have been made (see table below).

Yours sincerely,

Anna Karin Andersson

| **Reviewer's Responses to Questions** | **Revised version according to comments** |
| --- | --- |
| 4. Have the authors made all data underlying the findings in their manuscript fully available?  Reviewer #1: No | Due to the ethical approval and participants’ confidentiality, it is not possible to make the interviews fully available. However, we have added a table presenting an overview of the data analysis process from Meaning units to Core category. Also, a minimal data set is uploaded in the Supporting Information files. |
| **Reviewer’s comments to the Authors** | **Our answer** |
| 6. Reviewer #1: The authors addressed the concern regarding the rationale for studying preterm children nicely. This addition has helped to understand the perspective guiding the study. Incorporating studies of typically developing children does provide a basis for comparison of the information generated from the children born prematurely.  The inclusion of the theoretical perspective, the Family of Person-related Constructs, has been added to the introduction as requested and has enhanced the text.  Moving the ethical approval section to earlier in the text supports that this study was reviewed by the appropriate authorities prior to the data collection.  The authors addressed triangulation and trustworthiness of the methods without using those terms. Given that this is a qualitative study, it is important to include the terms addressing study rigor. The photographs and interviews are two different methods of data collection, thus address triangulation. Using multiple data analysts is another method of researcher triangulation. The efforts made by the authors to ensure that the study participants were able to take, choose, and discuss topics of their own choice does address trustworthiness. It will make the study stronger to include those specific terms. | Thank you for your comments.  The concepts of trustworthiness and triangulation is now added in Methods and Materials and in methodological considerations. Marked changes can be found in line 188-189, 397-399, 411-413, and in line 417. |
