# Supplementary material for: Meaningful everyday life situations from the perspective of children born preterm: A photo-elicitation interview study with six-year-old children
Source: PLoS One. 2023 Aug 14;18(8):e0284217. doi: 10.1371/journal.pone.0284217 (PMC10424858; doi:10.1371/journal.pone.0284217)
Supplement: Attachment — Submitted filename: PONE-D-22-19343R2 Review.docx [file pone.0284217.s006.docx]

Meaningful everyday life situations from the perspective of children born preterm: A photo-elicitation interview study with six-year-old children

PONE-D-22-19343R2

The authors provide appropriate justification for interviewing children to learn about what they find enjoyable and meaningful based on both previous studies and a theoretical model, the Family of Person Related Constructs (fPRC). The rationale for addressing children born prematurely is logical.

The informed consent process for both children and parents is well-described. A qualitative research design was used in the study incorporating data collection via interviews. The photo-elicitation method was used to elicit those situations that were meaningful to them, as well as to facilitate their involvement in the interviews used in the research process. Details regarding the interview process used were provided.

The data analysis processes used and the resulting codes were clearly documented. A few quotes were woven into the results section. More quotes would have supported the codes in more depth, but this is not a pressing concern.

A previous concern was the lack of information regarding qualitative rigor processes. That concern has been addressed in this paper, where there is discussion of triangulation in the data analysis process and the data collection process. The use of the photo-elicitation procedure and the strengths and limitations that procedure were well documented and related to the qualitative research process.

The manuscript has been strengthened and in my opinion, is appropriate for publication.
